# Rhizosphere soil microbial community and its response to different utilization patterns in the semi-arid alpine grassland of northern Tibet

**DOI:** 10.3389/fmicb.2022.931795

**Published:** 2022-07-22

**Authors:** Lijiao Fu, Yan Yan, Xueqin Li, Yanling Liu, Xuyang Lu

**Affiliations:** ^1^Key Laboratory of Mountain Surface Processes and Ecological Regulation, Institute of Mountain Hazards and Environment, Chinese Academy of Sciences, Chengdu, China; ^2^Chinese Academy of Sciences, University of the Chinese Academy of Sciences, Beijing, China

**Keywords:** rhizosphere soil microorganisms, community structure, diversity, interaction network, DNBSEQ sequencing, alpine steppe

## Abstract

As the link between plants and soils, rhizosphere soil microorganisms play an important role in the element cycle. This study aimed to understand the response of the rhizosphere soil microbial community structure and interaction network to grassland utilization in the alpine steppe of the northern Tibet Plateau. High-throughput sequencing was employed to study the composition, diversity, and species interaction network of rhizosphere soil microbial communities under grazing, mowing, and enclosing treatments. *Proteobacteria* (47.19%) and *Actinobacteria* (42.20%) were the dominant bacteria in the rhizosphere soil. There was no significant difference in relative abundance among rhizosphere soil microorganisms at phylum and genus levels, but differences were found in *Chlorobi, Ignavibacteriae*, and *Micromonospora*. The alpha diversity index based on Shannon, Chao1, and Simpson indices revealed that except for a significant difference in the Shannon index of the *Artemisia nanschanica* group, the richness and evenness of rhizosphere soil microbial communities among all groups were similar. Non-metric multidimensional scaling (NMDS) and multi-response permutation procedure (MRPP) analyses showed that the inter-group differences of three plants (*Stipa purpurea, Carex moorcroftii*, and *Artemisia nanschanica*) were greater than the differences within the groups; however, only the inter-group difference with the *Stipa purpurea* group was significant. The microbial interaction network showed that the network complexity of the *Artemisia nanschanica* group and the enclosing treatment, which were not easily influenced by external factors, were higher than those of the other groups and treatments; this again demonstrated that *Proteobacteria* and *Actinobacteria* were the network core microbial species in alpine steppe of the northern Tibet Plateau and were crucial for maintaining stability of the microbial communities. Findings from this study provide a theoretical basis for the restoration of degraded alpine grassland and the development of microbial functions.

## Introduction

Soil microorganisms are not only the predominant biological component in the ground but are the most active living beings in the soil system, with an irreplaceable role in soil formation and development, plant growth, and ecosystem stability (Powlson et al., 2001; Lin and Hu, 2008). An important function of soil microorganisms is to transform soluble and insoluble organic matter into inorganic forms that can be absorbed and utilized by plants for essential roles in growth and stress resistance. For example, organic matter can be reused by plants through the processes of oxidation, ammoniation, nitrification, nitrogen fixation, and vulcanization performed by microorganisms in the soil (Porazinska et al., 2003; Ju and Ting, 2007). Thus, soil microorganisms are of great significance to the cycling process of elements, such as C and N.

As a subsystem of soil microorganisms (Jin et al., 2009), rhizosphere microorganisms have important functions in plant colonization, growth, reproduction, and community succession (Lambers et al., 2009; Sun et al., 2017). Plant growth and development is affected by both the positive and negative impacts of rhizosphere microorganisms on vegetation. On the one hand, rhizosphere soil microorganisms can transform nutrients in the soil into a form that plants can absorb and utilize through their own metabolic activities, thereby maintaining the nutrient cycle of the ecosystem (Ai et al., 2015). In addition, some microbial populations, such as arbuscular mycorrhizal fungi, form symbiotic relationships with plants (Omirou et al., 2016). These mycorrhizal fungi obtain essential carbohydrates and other nutrients from plants, while the plants also acquire essential nutrients and water from the fungi. On the other hand, some rhizosphere soil microorganisms compete for soil resources with plants, that is, there is a balance between the absorption of soil nutrients by vegetation and the retention of nutrients by microorganisms. In addition, the occurrence of plant diseases has been reported to be related to the changes in soil microbial flora and diversity (Mendes et al., 2011; Pfeiffer et al., 2017). Increases in the number of pathogenic bacteria among soil microorganisms leads to vegetation infection and disease, even death of vegetation, which is not conducive to plant growth and development. The rhizosphere soil microbial community structure and diversity can therefore reflect not only the composition and functional diversity of an ecosystem but also the health state of vegetation, which is vital for the feedback regulation of soil microorganisms and vegetation.

Early grassland studies predominantly focused on soil physical and chemical properties (Hou and Ren, 2003), vegetation community structure (Zhang et al., 2019b), coverage (Quan et al., 2015), species composition, and aboveground/underground biomass (Yan and Lu, 2015). The development of high-throughput sequencing technology (Shendure and Ji, 2008) led to increasing attention on the changes of soil microorganisms (Ren et al., 2018; Li et al., 2020b; Qu et al., 2021). Different grassland management measures can result in changes in soil microbial communities (Wang et al., 2020b). For example, Zhao et al. (2017) found that grazing significantly reduced the number of soil microorganisms; Zhang et al. (2014) and Wei et al. (2020) found that grassland mowing had little effect on soil microbial community diversity; and Yin et al. (2019) found that long-term enclosure had no significant effect on soil microorganisms in mildly degraded grassland. In addition, a grazing experiment in Inner Mongolia showed that heavy grazing reduced the number of soil microorganisms in the rhizosphere of *Artemisia frigida*, while moderate grazing increased the number of microorganisms (Zhang et al., 2017). Another study in Inner Mongolia showed that both grazing and mowing reduced the number of microorganisms in the rhizosphere, but the influence of grazing was greater than that of mowing (Hu et al., 2015). Furthermore, a cutting experiment in the Ordos Plateau showed that cutting had no significant effect on the total amount of microorganisms in rhizosphere soil but significantly affected the composition of bacteria, fungi, and actinomycetes (Zhang et al., 2016b). At present, researchers studying plant rhizosphere microorganisms tend to compare and analyze the differences between plant rhizosphere microorganisms and non-rhizosphere microorganisms or focus on the effects of a certain treatment on plant rhizosphere microorganisms. There is also a lack of research on the effect of common grassland utilization measures on plant rhizosphere soil microorganisms, especially some typical dominant species.

Northern Tibet, located in the hinterland of the Qinghai–Tibet Plateau, is rich in grassland resources and is the production base of animal husbandry in Tibet Autonomous Region, as well as an important ecological security barrier area in China (Gao et al., 2010). Alpine steppe is the most widely distributed grassland type in northern Tibet, accounting for about two-thirds of the grassland on the Qinghai–Tibet Plateau, and it has good ecosystem service value. However, the alpine grassland ecosystem is extremely fragile due to harsh natural conditions, sensitivity of the area to climate change, and the increase in human activities in recent years (Harris, 2010; Zhang et al., 2019b). Numerous studies investigating the reasons underlying the fragility of this ecosystem have concluded that grazing is the predominant driving force leading to the changes of alpine grassland (Li et al., 2019; Wang et al., 2020a). Furthermore, in terms of the management and restoration of degraded grassland, most studies show that grazing prohibition can improve the quality of degraded grassland and is an effective measure to control grassland degradation (Chen et al., 2014; Yan and Lu, 2015).

Grazing and mowing are the two main systems of grassland management, and enclosing is an effective measure for the restoration of degraded grassland. Although there have been extensive studies on the effects of different utilization patterns on the plant community structure and soil microbial communities, limited attention has been paid to the effects of different utilization patterns on rhizosphere soil microbial communities in different plants. Therefore, in the current study, an experiment featuring three different grassland utilization methods of enclosing, grazing, and mowing was designed. Subsequently, differences in the microbial community structure and composition in the rhizosphere soil of three dominant plants under different utilization patterns in the semi-arid habitat of alpine steppe in the northern Tibet Plateau were explored. The main goal of this study was to investigate whether the response of the rhizosphere microbial community structure, diversity, and interactions of different plants under different grassland utilization patterns was consistent.

## Materials and methods

### Study site

**Figure 1:**
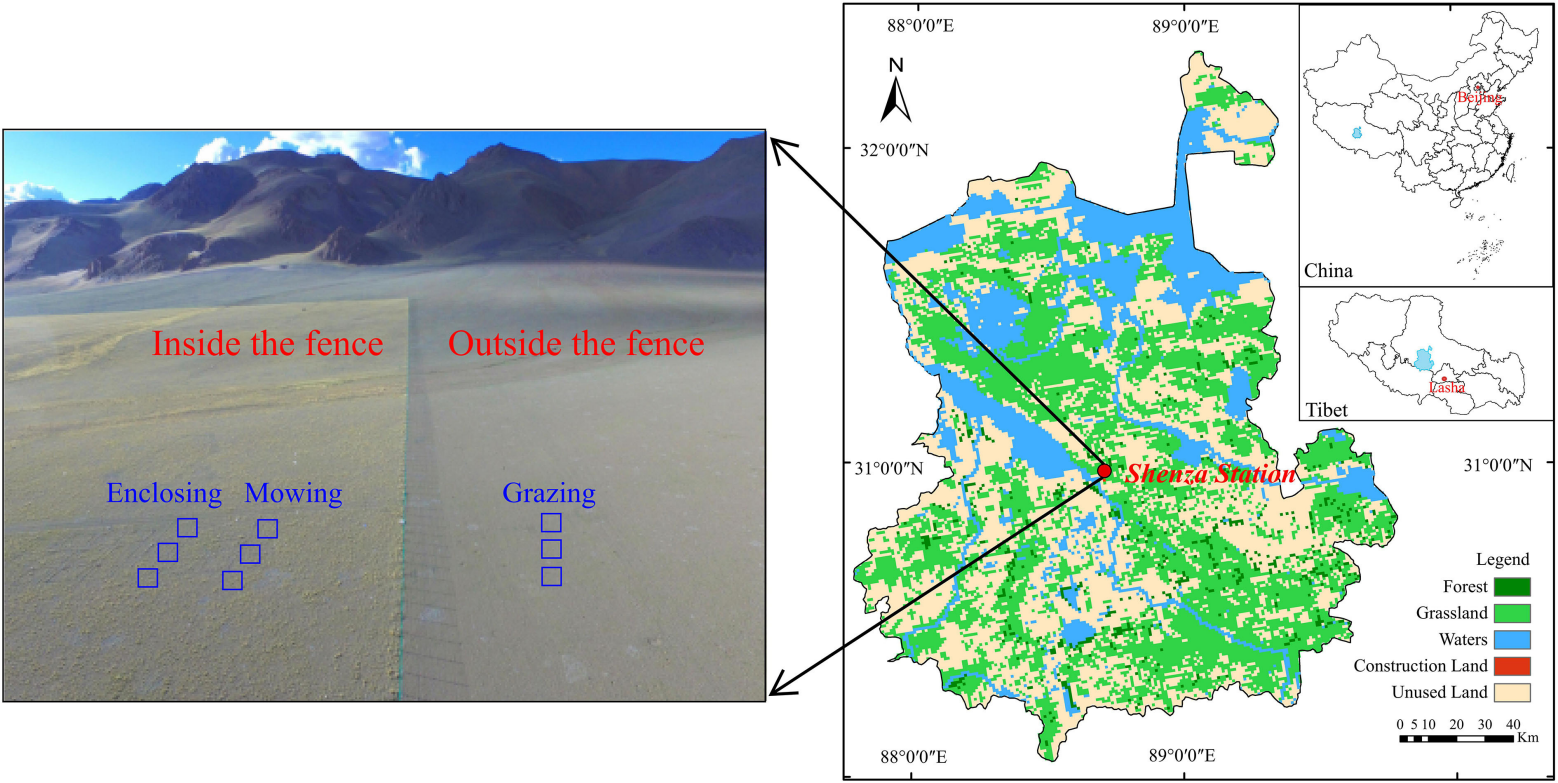
Location of the study area and experimental layout inside and outside the fence.

The experiment was conducted in the permanent sample plot of the Shenza alpine steppe and the Wetland Ecosystems Observation and Experimental Station (30°57′ N, 88°42′ E, 4,675 m elevation, hereinafter referred to as Shenza Station; Figure 1) of the Institute of Mountain Hazards and Environment, Chinese Academy of Sciences, in Shenza County, Nagqu city, Tibet Autonomous Region. This region is very eco-environmentally autochthonous and well-preserved in its original traits, making it an ideal location to observe the rhizosphere microorganisms of dominant plants in alpine steppe. The Shenza Station is in the alpine steppe of the northern Tibet Plateau, the hinterland of the Qinghai–Tibet Plateau. The climate belongs to the semi-arid monsoon climate region of the sub-cold zone of the plateau, with thin and cold, dry air. The average annual temperature is 0.4°C, the annual sunlight duration is 2,915 h, and annual frost duration is 279 h. The region experiences perennial drought with little rain and uneven distribution, and the mean annual precipitation is 298.6 mm, which mainly occurs from May to September. The vegetation type of the area belongs to alpine steppe, and the community structure of the vegetation is relatively simple, with the dominant species being *Stipa purpurea*, accompanied by *Carex moorcroftii, Artemisia nanschanica, Poa annua, Stellera chamaejasme, Oxytropis microphylla*, and *Leontopodium nanum*. Vegetation in the area greens in the middle to late May of each year grows from June to August, gradually stops growing in September, and starts withering in October (Yan et al., 2014). As a pure animal husbandry area, grazing is the main source of income for herdsmen in northern Tibet.

### Experimental design

The experiment was conducted inside and outside the fence of experimental land of alpine grassland at Shenza Station in 2021 (Figure 1). A total of nine plots, each 3 × 3 m, were established, of which six were inside the fence and the other three were outside. Grassland within the fence had been enclosed in 2014, and of the six plots in the fenced area, three were used for mowing and the other three served as the enclosed area without treatment. Mowing was carried out in the middle of the growing season (July) at 1–2 cm above the ground, and all plants were mowed. The three plots set outside the fence were meadows with local herders performing grazing activities, and the grazing livestock were predominantly yaks. The interval between plots under each treatment was 2 m. Samples were taken at the end of the growing season (September).

The top three dominant species in alpine grassland—*Stipa purpurea* (family Gramineae), *Carex moorcroftii* (family Cyperaceae), and *Artemisia nanschanica* (family Compositae)—were selected as research objects. Totally, 10 individual plants were randomly selected from each sample plot, and their roots were dug out completely. Loose bulk soil was shaken from the roots, and then the soil attached to the root surface (about 1 mm) was collected with a sterilized brush and placed into a 10-mL sterile tube as a rhizosphere soil sample, and the procedure and repeated three times. A total of 27 rhizosphere soil samples were collected in the experiment.

### DNA extraction and DNBSEQ sequencing of rhizosphere soil samples

First, a 96-well deep-hole plate was prepared to load swabs from the rhizosphere soil. DNA from each rhizosphere soil sample was extracted by using the magnetic beads method, utilizing the Kingfisher purification system to automatically extract and purify DNA. At the end of the program, the DNA solution in the deep-hole plate was transferred to a 1.5-mL centrifuge tube for storage. Subsequently, the DNA solution was used for library construction and sequencing on the DNBSEQ sequencing platform.

The main steps for the construction of the DNBSEQ sequencing platform library are as follows: (1) Sample testing, including the concentration, integrity, and purity of the sample. The concentration was determined by fluorescence quantification or enzyme-labeling instrument. The integrity and purity of the sample were tested by agarose gel electrophoresis (agarose gel concentration: 1%, voltage: 150 V, electrophoresis time: 40 min). (2) Sample interruption: 1 μg genomic DNA was interrupted by ultrasound with a Covaris instrument. (3) Fragment size selection: The interrupted sample was selected by magnetic beads to concentrate the sample at approximately 200–400 bp. (4) End repair: adding “A” base for joint connection. (5) Preparation of the reaction system: React at suitable temperature for a certain time, repair the end of double-stranded cDNA, add A base to the 3′ end, prepare the joint connection reaction system, and react at suitable temperature for a certain time to make the joint connect with DNA. (6) PCR and product recovery: The PCR system was prepared, and the reaction procedure was set up to amplify the linked products. Amplified products were subsequently purified and recovered by magnetic beads. (7) Product cyclization: The PCR product was denatured into a single chain, the cyclization reaction system was prepared, and the single-chain ring product was obtained by fully mixing the reaction at the right temperature for a certain time. The final library was obtained after digesting the uncyclized linear DNA molecules. (8) Library detection: The concentration of the cyclization product was detected before going on the machine. (9) Generation of sequencing data: The qualified library was arranged for computer sequencing (DNBSEQ): single-stranded circular DNA molecules were copied through rolling rings to form a DNA nanoball (DNB) containing multiple copies. DNBs were added to the reticular pores of the chip using high-density DNA nano-chip technology and were sequenced by joint probe anchoring polymerization.

### Processing of sequencing data

Original sequences obtained from the DNBSEQ sequencing platform were filtered by Soap2 (Li et al., 2009) to remove reads with low quality, joint contamination, and high unknown base N content. The resulting high-quality short clean reads of each DNA sample were assembled by MEGAHIT (Li et al., 2015). MetaGeneMark (Zhu et al., 2010) was used for the prediction of metagenomic genes. The assembled reads were clustered to remove redundancy by CD-hit (Fu et al., 2012), and finally, unigenes were obtained (the similarity threshold is 95%). Salmon software (version1.4.0) was used to standardize sequences through the transcripts per million (TPM) method to determine gene abundance. Species annotation and species abundance were calculated with Kraken (Wood and Salzberg, 2014).

### Statistical analyses

The alpha diversity of rhizosphere soil microbial communities was calculated by QIIME (version1.80) (Caporaso et al., 2010). The formula for calculating the diversity indices are as follows:


(1) $$H_{Shannon}=-\sum A_{i}^{*}\ln(A_{i})$$

(2) $$D_{Simpson}=\frac{\sum_{i=1}^{S_{obs}}n_{i}(n_{i}-1)}{N(N-1)}$$

(3) $$\begin{array}{r} S_{Chao1}=S_{obs}+\frac{F_{1}\left(F_{1}-1\right)}{2\left(F_{2}+1\right)} \end{array}$$

In the formulae, A_i_ is the relative abundance of species i; N is the total number of individuals in all species; n_i_ represents the total number of individuals of species i; S_obs_ is the number of species observed in the sample; and F_1_ and F_2_ represent the number of singletons and doubletons, respectively.

The Kruskal–Wallis test in non-parametric statistics, realized by SPSS25.0 (IBM, Chicago, IL, USA), was used to analyze the difference of relative abundance of the top 15 species in the rhizosphere soil microorganisms at the phylum and genus levels under different treatments, as well as the alpha diversity of rhizosphere soil microorganisms. All other statistical analyses were performed in the R environment (version 4.1.3). The relative abundance of the top 30 species at the phylum and genus levels was used to show the overall situation of rhizosphere soil microorganisms under different grassland utilization patterns. The random forest model (“randomForest” package, Version 4.7–1) (Breiman, 2001) was employed to determine indicator species at the phylum and genus levels. Based on the Bray–Curtis dissimilarity calculated by “vegan” package (version 2.5–7) (Dixon, 2003), non-metric multidimensional scaling (NMDS) (Kenkel and Orloci, 1986), and multi-response permutation procedure (MRPP) analyses were used to evaluate the beta diversity of rhizosphere soil microbial communities. The alpha diversity violin chart was drawn by “vioplot” package (Version 0.3.7) (Hu, 2020), while other graphs were generated through the “ggplot2” package (Version 3.3.5) (Wickham, 2009).

The microbial species network was constructed based on data of species with >0.1% average relative abundance of microbial genera in the rhizosphere soil of three plants. First, the “psych” package (version 2.2.3) (Revelle and Condon, 2019) in R was used to analyze the network between species. The correlation coefficient was standardized by the Spearman correlation analysis method and false discovery rate method, and the related species data of |r|>0.7 and *P* < 0.05 were reserved to construct the correlation network. Next, network visualization analysis was performed using Gephi (version 0.9.2). Processed network correlation data were imported into Gephi, the layout was conducted by using the Frucherman–Reingold algorithm, and the network topology parameters, such as characteristic path length, number of edges, number of nodes, average clustering coefficient, network density, and average connectivity, were obtained.

The map displaying geographical locations of the study area was constructed based on the remote sensing monitoring data of land use in Tibet in 2020 downloaded by the Resource and Environmental Science and Data Center and the software ArcGIS (version 10.5, Environmental Systems Research Institute, Inc., CA, USA).

## Results

### Rhizosphere soil microbial community structure

**Figure 2:**
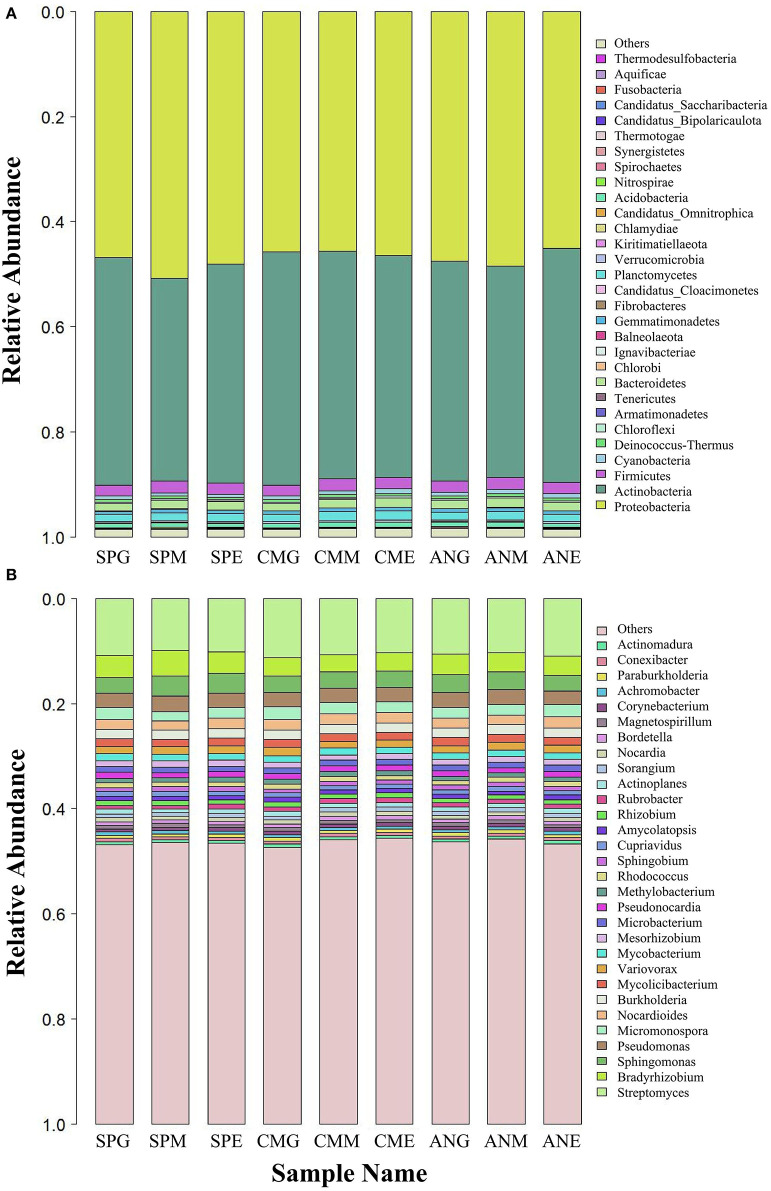
Relative abundance of the top 30 rhizosphere soil microorganisms at phylum **(A)** and genus **(B)** levels under different grassland utilization patterns. SPG, *Stipa purpurea* group under grazing treatment; SPM, *Stipa purpurea* group under mowing treatment; SPE, *Stipa purpurea* group under enclosing treatment; CMG, *Carex moorcroftii* group under grazing treatment; CMM, *Carex moorcroftii* group under mowing treatment; CME, *Carex moorcroftii* group under enclosing treatment; ANG, *Artemisia nanschanica* group under grazing treatment; ANM, *Artemisia nanschanica* group under mowing treatment; ANE, *Artemisia nanschanica* group under enclosing treatment.

**Figure 3:**
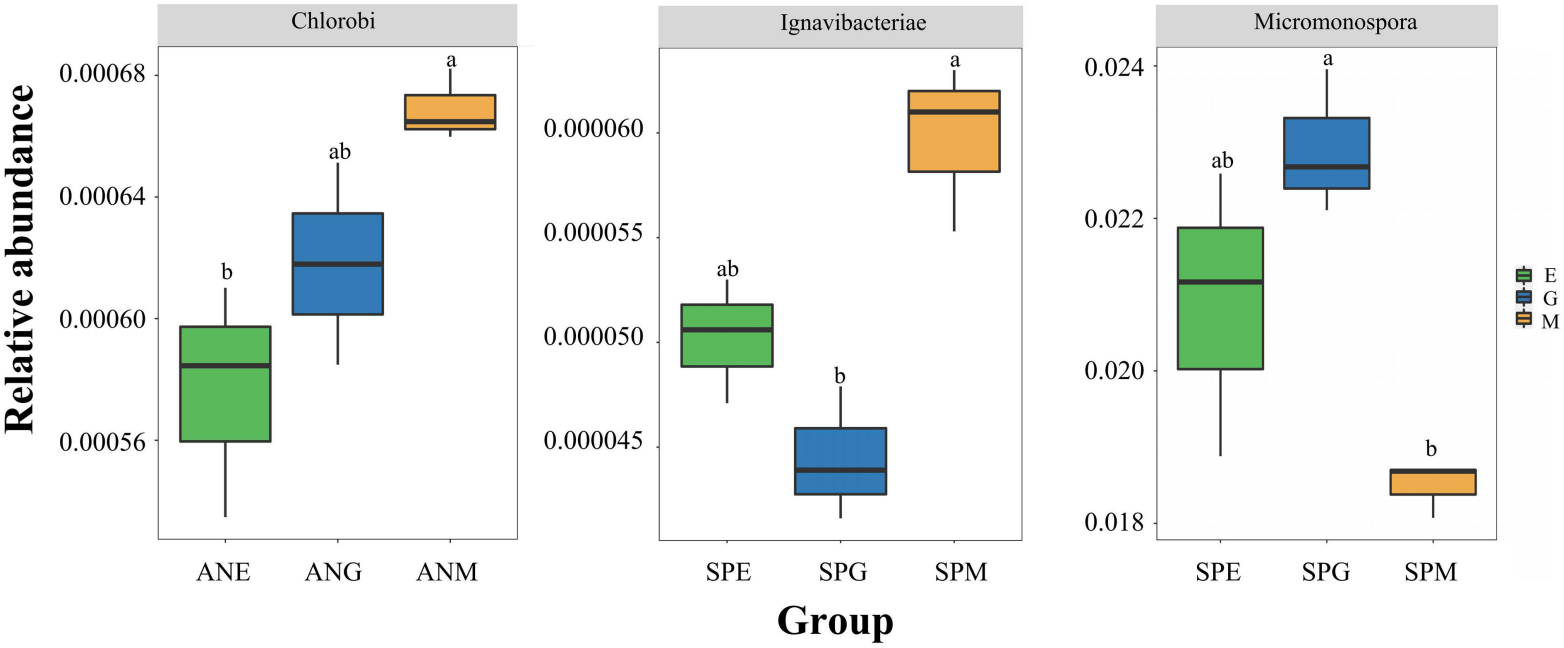
Pairwise comparison of different rhizosphere soil bacteria at phylum and genus levels in each group. Different lowercase letters indicate that the relative abundance of rhizosphere bacteria of the same plant is significantly different (*P* < 0.05) under different utilization patterns.

At the phylum level (Figure 2A), top 15 rhizosphere soil microorganisms ranked by relative abundance were *Proteobacteria, Actinobacteria, Firmicutes, Cyanobacteria, Deinococcus-Thermus, Chloroflexi, Armatimonadetes, Tenericutes, Bacteroidetes, Chlorobi, Ignavibacteriae, Balneolaeota, Gemmatimonadetes, Fibrobacteres*, and *Candidatus Cloacimonetes*. Among them, *Proteobacteria* and *Actinobacteria* were the dominant phyla, with an average relative abundance of 47.19 and 42.20%, respectively. Kruskal–Wallis tests (Table 1) revealed only *Chlorobi* and *Ignavibacteriae* showed a significant difference (*P* < 0.05) between groups; there were no significant differences between groups among all other bacteria. Pairwise comparison of rhizosphere soil bacteria with significant differences in relative abundance under different grassland utilization patterns showed that *Chlorobi* in the *Artemisia nanschanica* group under mowing treatment was significantly higher than that in the enclosed area (Figure 3). This was also consistent with the difference of *Ignavibacteriae* in the *Stipa purpurea* group (Figure 3).

**Table 1:** Kruskal-Wallis test of the top 15 rhizosphere soil microorganisms under different grassland utilization patterns at phylum level.

**Phylum**	**Group**		
	**SPG-SPM-SPE**	**CMG-CMM-CME**	**ANG-ANM-ANE**
*Proteobacteria*	0.061	0.957	0.177
*Actinobacteria*	0.051	0.670	0.079
*Firmicutes*	0.252	0.177	0.329
*Cyanobacteria*	0.491	0.113	0.733
*Deinococcus-Thermus*	0.587	0.957	0.202
*Chloroflexi*	0.733	0.148	0.252
*Armatimonadetes*	0.430	0.301	0.113
*Tenericutes*	0.177	0.113	0.066
*Bacteroidetes*	0.066	0.118	0.113
*Chlorobi*	0.252	0.430	0.039
*Ignavibacteriae*	0.039	0.118	0.099
*Balneolaeota*	0.561	0.301	0.066
*Gemmatimonadetes*	0.733	0.957	0.252
*Fibrobacteres*	0.733	0.051	0.088
*Candidatus_Cloacimonetes*	0.113	0.288	0.957

At the genus level (Figure 2B), species with an average relative abundance of more than 1% were defined as dominant microorganisms, and these accounted for about 30% of the relative abundance of all classifications. The microorganisms from highest to lowest according to relative abundance were *Streptomyces, Bradyrhizobium, Sphingomonas, Pseudomonas, Micromonospora, Nocardioides, Burkholderia, Mycolicibacterium, Variovorax, Mycobacterium, Mesorhizobium, Microbacterium*, and *Pseudonocardia*. Kruskal–Wallis tests (Table 2) revealed that while there were no significant differences among most microorganisms in *Stipa purpurea, Carex moorcroftii*, and *Artemisia nanschanica* groups, there was a significant difference with *Micromonospora* in the *Carex moorcroftii* group, with a *P*-value of 0.039. This indicated that there were statistical differences in rhizosphere soil microorganisms under different grassland utilization patterns (*P* < 0.05). Pairwise comparison of rhizosphere soil bacteria *Micromonospora* under different grassland utilization patterns showed that *Micromonospora* under grazing treatment was significantly higher than that under mowing treatment (Figure 3), and there was no significant difference between the other two groups.

**Table 2:** Kruskal-Wallis test of the top 15 rhizosphere soil microorganisms under different grassland utilization patterns at genus level.

**Genus**	**Group**		
	**SPG-SPM-SPE**	**CMG-CMM-CME**	**ANG-ANM-ANE**
*Streptomyces*	0.177	0.329	0.113
*Bradyrhizobium*	0.061	0.837	0.561
*Sphingomonas*	0.066	0.875	0.113
*Pseudomonas*	0.252	0.733	0.079
*Micromonospora*	0.039	0.288	0.202
*Nocardioides*	0.079	0.561	0.393
*Burkholderia*	0.670	0.733	0.113
*Mycolicibacterium*	0.393	0.837	0.288
*Variovorax*	0.430	0.561	0.733
*Mycobacterium*	0.193	0.837	0.301
*Mesorhizobium*	0.066	0.491	0.561
*Microbacterium*	0.061	0.148	0.058
*Pseudonocardia*	0.051	0.561	0.177
*Methylobacterium*	0.393	0.193	0.733
*Rhodococcus*	0.099	0.561	0.288

**Figure 4:**
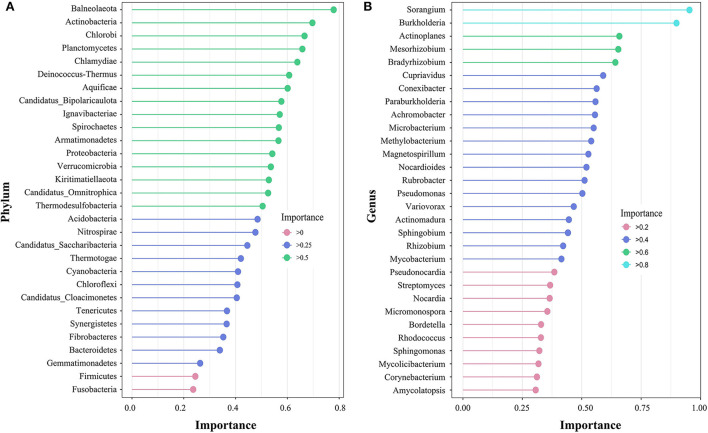
Species importance point map according to the importance of the top 30 rhizosphere soil microorganisms at the phylum **(A)** and genus **(B)** levels on the northern Tibetan alpine steppe.

The random forest model can show indicator species with high importance values. At the phylum level (Figure 4A), the importance value of the top 30 species was between 0.2 and 0.8, and no species presented obvious importance. However, at the genus level (Figure 4B), *Sorangium* and *Burkholderia* were extremely sensitive indicator bacteria (importance >0.8) in the alpine steppe of Shenza.

### Alpha diversity of rhizosphere soil microbial community

**Figure 5:**
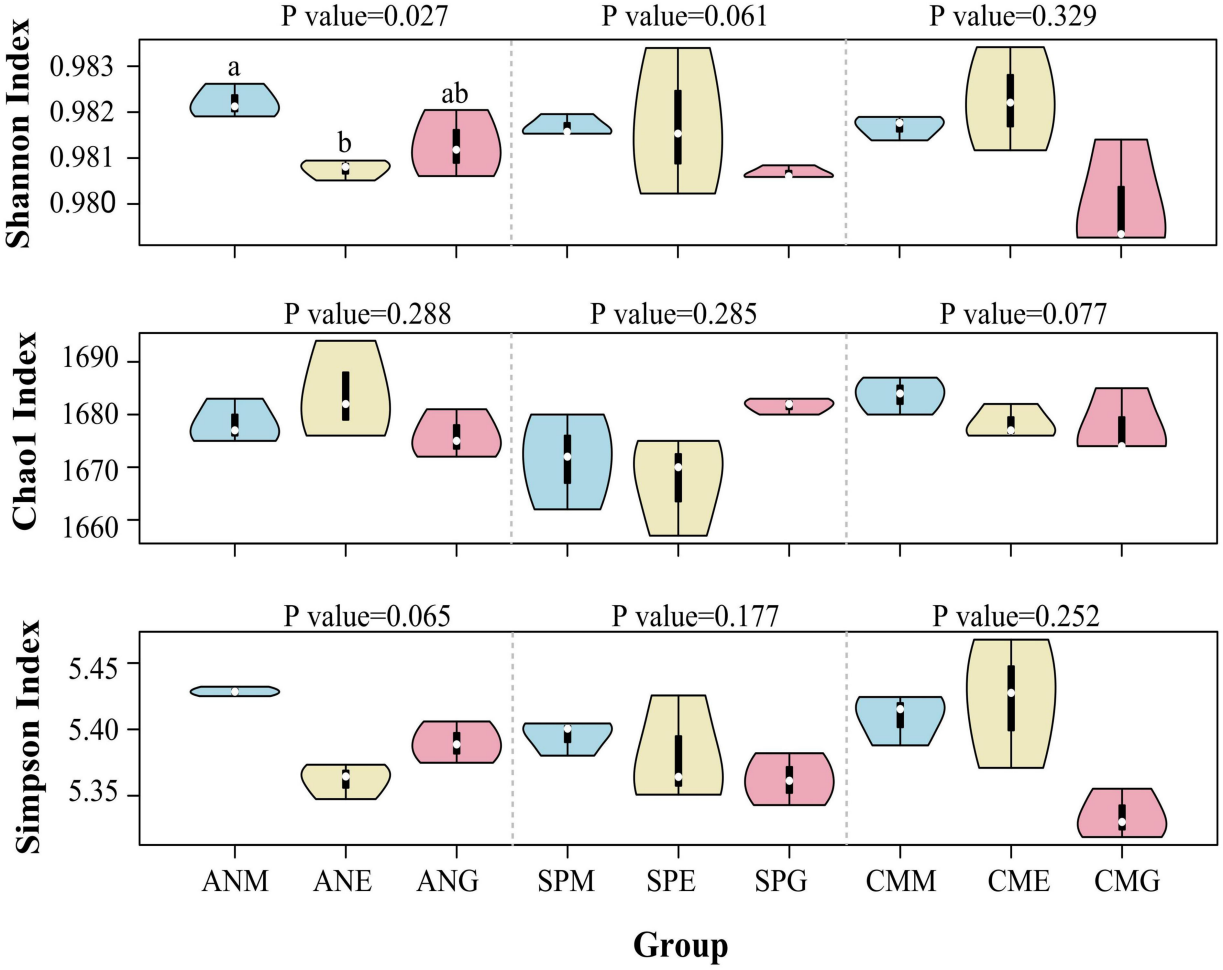
Violin diagram based on alpha diversity indices. Groups with significant differences are marked with letters, while groups without a letter did not show significant differences.

The alpha diversity violin map (Figure 5) based on Shannon, Chao1, and Simpson indices showed that the Shannon index of all samples was >0.97, with the Shannon index of *Artemisia nanschanica* under mowing treatment significantly higher than that of *Artemisia nanschanica* under enclosing treatment (*P* < 0.05). Differences of other alpha diversity indices in different grassland utilization patterns were not significant (*P* > 0.05), indicating that the alpha diversity of the three plants surveyed in different grassland utilization patterns was similar.

### Beta diversity of rhizosphere soil microbial community

**Figure 6:**
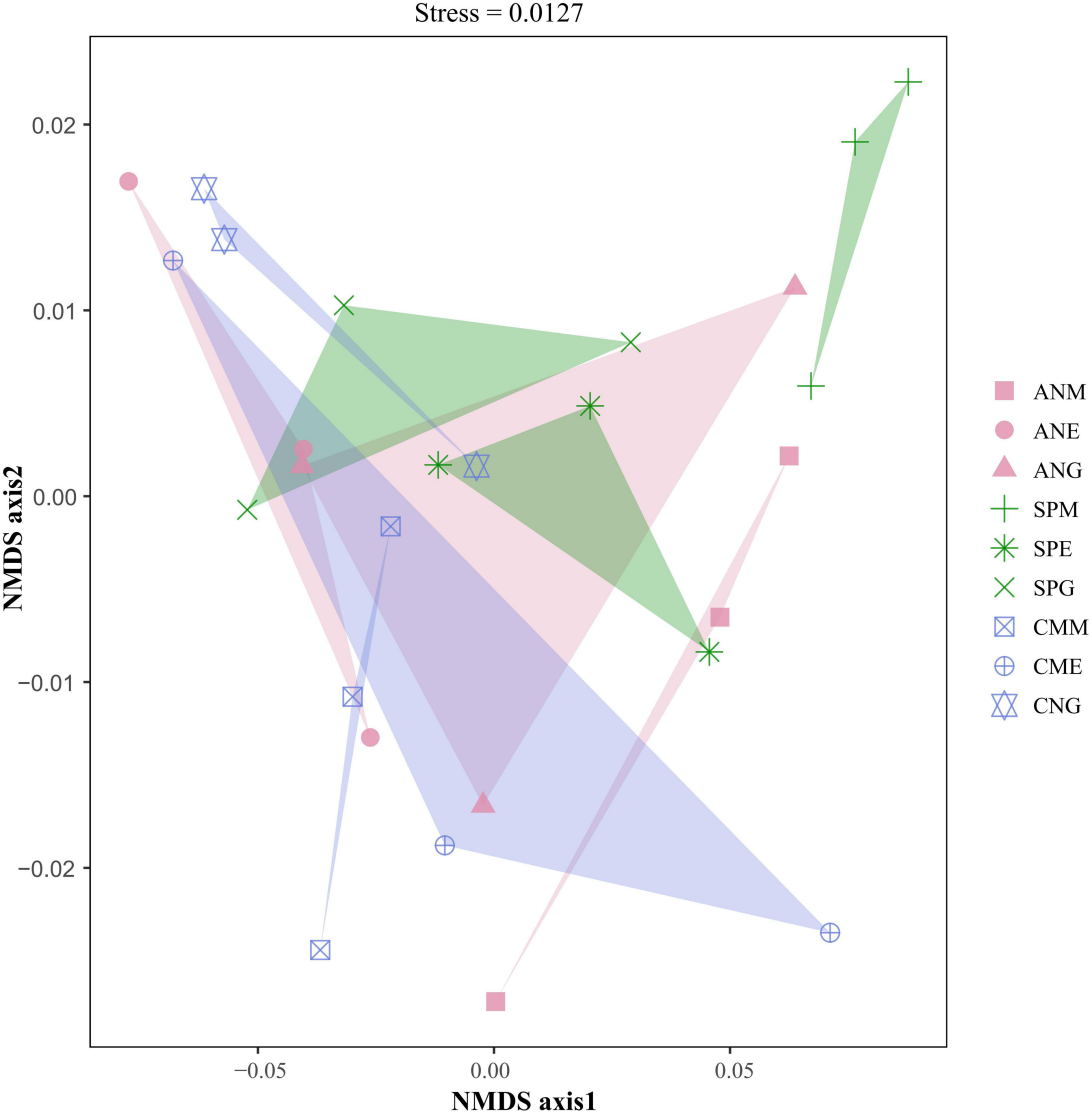
Non-metric multidimensional scaling (NMDS) analysis of the rhizosphere soil microorganisms in alpine grasslands on the northern Tibetan alpine steppe. Different colors represent different plants.

NMDS based on Bray–Curtis dissimilarity was used to reflect the microbial beta diversity in plant rhizosphere soil under different utilization patterns of Shenza alpine steppe (Figure 6). The rhizosphere microbial communities of the three plants were significantly separated (stress = 0.0127) under different grassland utilization patterns. For example, there were significant differences in Bray–Curtis dissimilarity among *Stipa purpurea* under grazing, mowing, and enclosing treatments, indicating that *Stipa purpurea* had significant differences in rhizosphere soil microbial communities under different grassland utilization patterns. In addition, the Bray–Curtis dissimilarity between the *Artemisia nanschanica* group under mowing and enclosing treatments, and the *Carex moorcroftii* group under grazing and enclosing treatments was close, indicating that the microbial community structure in the rhizosphere soil between them was similar at the phylum level.

MRPP emphasized the intra- and inter-group differences of microbial communities in plant rhizosphere soil under different grassland utilization patterns (Table 3). The A values of the three plants were all >0, indicating that the differences between groups were greater than the differences within groups. Furthermore, the inter-group difference was significantly greater than the intra-group difference in the *Stipa purpurea* group (*P* < 0.05).

**Table 3:** Multi-response permutation procedure (MRPP) analysis of differences among groups.

**Group**	**A**	**Observe delta**	**Expect delta**	***P*** **-value**
SPG-SPM-SPE	0.337	0.022	0.033	0.038
CMG-CMM-CME	0.008	0.037	0.038	0.398
ANG-ANM-ANE	0.135	0.034	0.040	0.105

*An A value >0 means that the difference between groups is greater than that within groups, while an A value <0 means that the difference between groups is less than that within groups. Expect delta value represents the magnitude of inter-group differences and Observe delta value represents the magnitude of intra-group differences. A P < 0.05 indicates that the difference is significant*.

### Microbial interaction network

**Figure 7:**
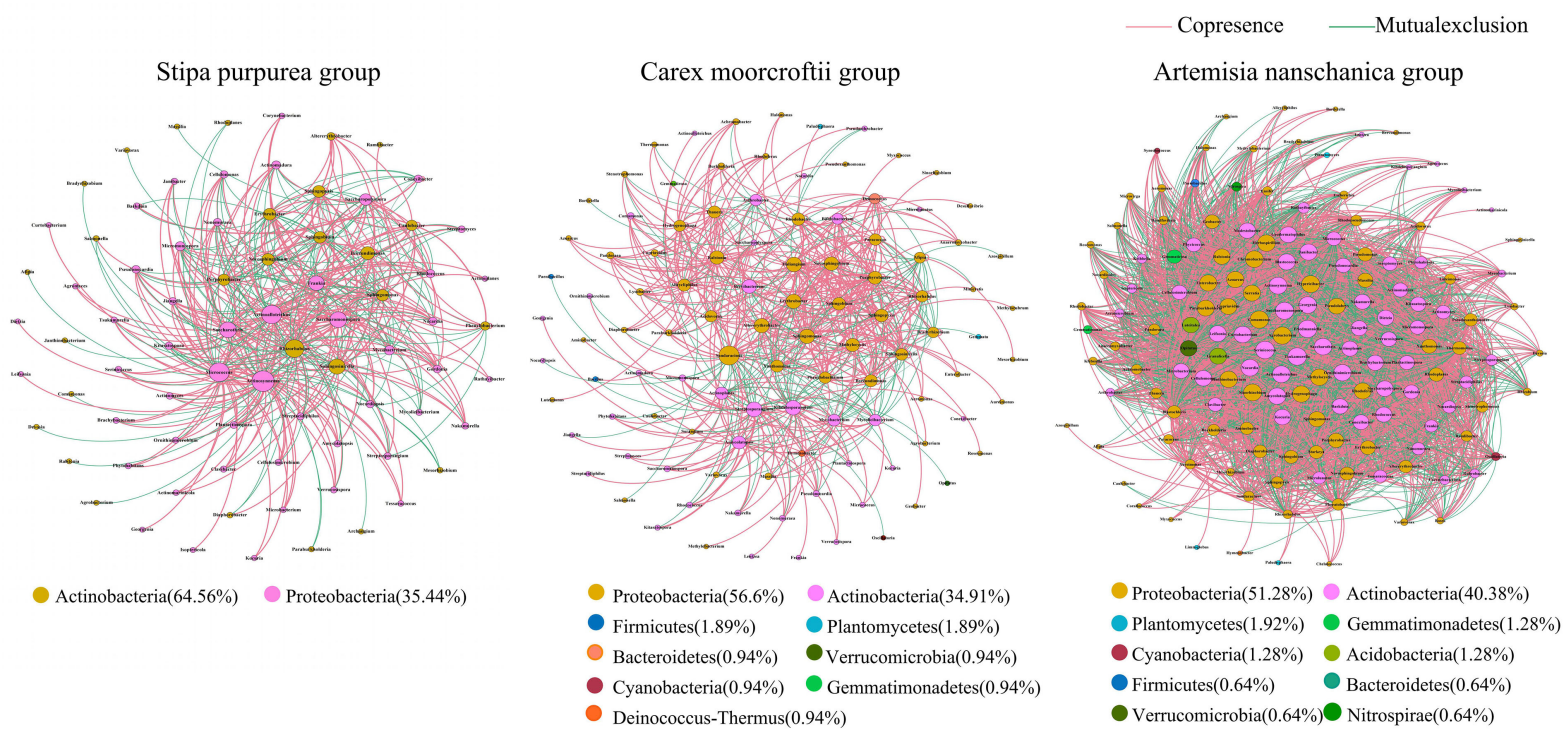
Relationship network of rhizosphere soil microbial communities among different plants. The size of nodes in the figure is proportional to the degree of network nodes, and nodes are colored according to different phyla. The color of the edge between the node and the node shows the interaction between species: red indicates a positive relationship, and green indicates a negative relationship. The width of the edge and the correlation coefficient |r| value are proportional.

**Figure 8:**
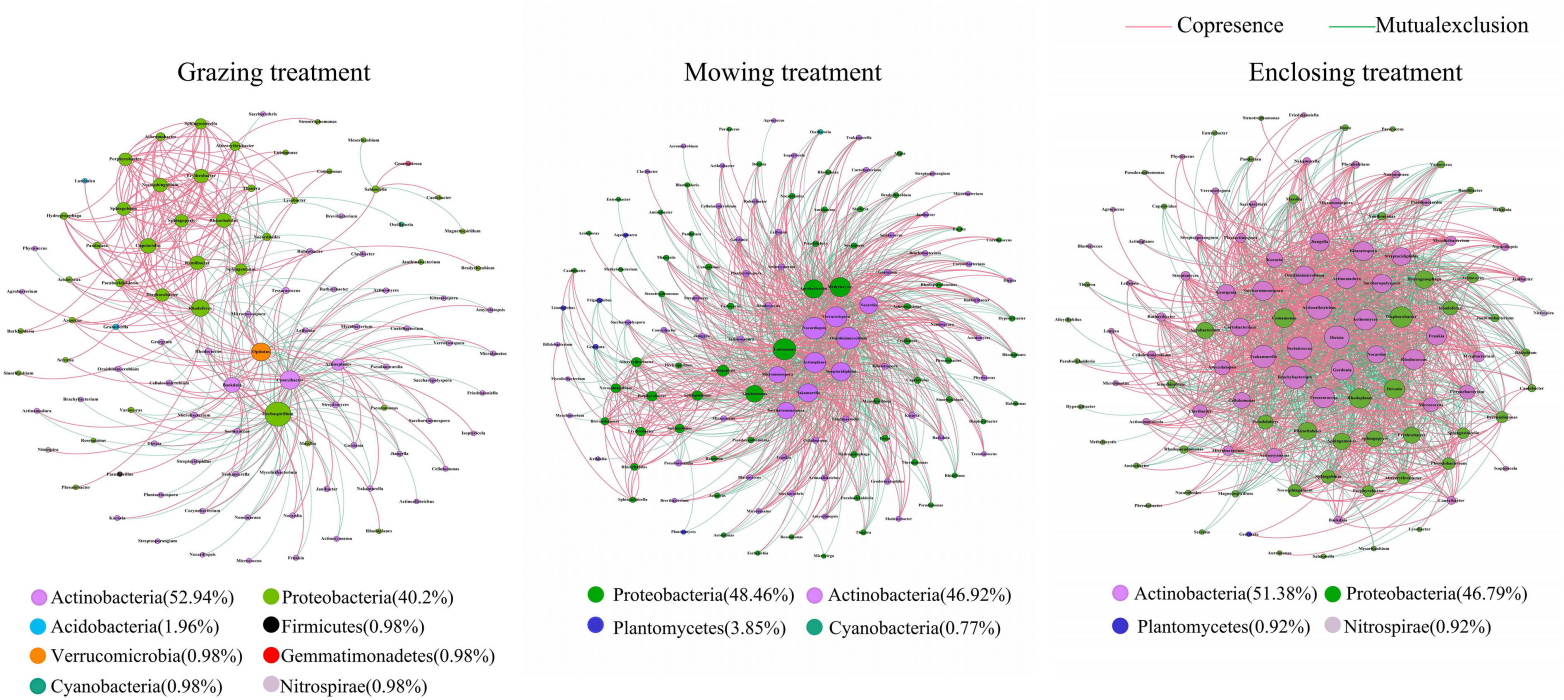
Relationship network of rhizosphere soil microbial communities under different utilization patterns. The explanation of the figure is the same as that of Figure 7.

Based on the relative abundance data of microbial genera in rhizosphere soil of different plants (Figure 7) and different utilization patterns (Figure 8), the microbial community interaction network was constructed with the same similarity threshold of 0.7 under different groups, so that the topological structure coefficients of different networks could be displayed directly (Table 4). In the soil microbial community interaction network of three kinds of plants (Figure 7), the average degree, the number of nodes, and the number of edges were highest in the *Artemisia nanschanica* group, followed by the *Carex moorcroftii* group, and then the *Stipa purpurea* group. The number of edges in the *Artemisia nanschanica* group was 5,285, which was ~12 times that of the *Stipa purpurea* group, and the average degree was approximately six times that of the *Stipa purpurea* group and the *Carex moorcroftii* group. This indicated that the relationship between soil microorganisms in the rhizosphere of *Artemisia nanschanica* was more complex, with the positive correlation accounting for 52.15% and the negative correlation accounting for 47.85%. In the *Carex moorcroftii* group, the proportion of positive correlation edges was 69.97% and the negative correlation edges was 30.03%, indicating that symbiotic relationships were dominant among microorganisms in the rhizosphere soil of *Carex moorcroftii*. The bacteria corresponding to the key species were predominantly *Actinobacteria* (51~65%) and *Proteobacteria* (34~41%) in all three plants. In general, the correlation among microbial communities was mainly positive, the key bacteria were *Actinobacteria* and *Proteobacteria*, and the relationship between the microbial communities in the rhizosphere soil of *Artemisia nanschanica*, which was not eaten by livestock, was the most complex among all those in the study.

**Table 4:** Topological index of rhizosphere soil microbial community interaction network among different plants and different utilization patterns.

**Network property**	**Plant group**			**Treatment group**		
	***Stipa purpurea***	***Carex moorcroftii***	***Artemisia nanschanica***	**Grazing**	**Mowing**	**Enclosing**
Clustering coefficient	0.815	0.742	0.818	0.703	0.885	0.804
Graph density	0.143	0.109	0.435	0.073	0.135	0.311
Average path length	2.082	2.440	1.657	2.508	2.065	1.952
Number of nodes	79	106	156	102	130	109
Number of edges	441	606	5,258	377	1,129	1,833
Positive edges	59.41%	69.97%	52.15%	69.76%	54.65%	57.67%
Negative edges	40.59%	30.03%	47.85%	30.24%	45.35%	42.33%
Average degree	11.165	11.434	67.410	7.392	17.369	33.633

In the interaction network of the rhizosphere soil microbial community under three different utilization patterns (Figure 8), the average degree and the number of edges were highest in the enclosing treatment, followed by the mowing treatment, and were lowest in the grazing treatment (Table 4). This indicated that the interrelationships among rhizosphere soil microbial genera in the enclosing area, which is not affected by external factors, were more complex. In addition, the proportion of positively correlated edges in the rhizosphere soil microbial community interaction network was larger than that of negatively correlated edges under the three different utilization patterns. This implied that the correlation between alpine grassland rhizosphere soil microbial community was mainly positive, and the corresponding phyla of key bacterial genera were *Actinobacteria* and *Proteobacteria*.

## Discussion

### Rhizosphere soil microbial community structures

The composition of rhizosphere microorganisms in grassland ecosystems is extremely complex (Wang et al., 2022). In the current study, the dominant bacteria at the phylum level were *Proteobacteria* and *Actinobacteria*, accounting for ~90% of the total microbial community abundance, while the secondary dominant bacterial phylum was Firmicutes, with a relative abundance of only 2% (Figure 2A). *Proteobacteria* and *Actinobacteria* are dominant bacteria not only in grassland (Zeng et al., 2017; Nan et al., 2020) but also in the process of secondary succession of abandoned cropland (Zhang et al., 2016a). However, the relative abundance of these phyla is quite different, which may be caused by different research areas and ecosystem types. For example, Ding et al. (2013) found that *Actinobacteria* was more dominant in arid and semi-arid habitats than in agricultural habitats. This was confirmed by Knelman et al. (2015), who reported that the structure of rhizosphere soil bacterial communities changes with changes in geographical distribution. Na et al. (2018) found that the smaller the geographical distance, the more similar the bacterial rhizosphere community structure, and the diversity and richness of the bacterial rhizosphere communities significantly and positively correlated with soil pH. In addition, comparison of soil *Chlorobi* in different vegetation zones in the middle reaches of Xilingol basin revealed that vegetation types influence the spatial distribution heterogeneity of soil *Chlorobi* microbial communities, and the status of plant cover has an indirect effect (Wang et al., 2021). This is contrary to the results of the present study, which showed that different grassland utilization patterns (mowing, enclosing, and grazing treatments inevitably lead to differences in vegetation cover) had significant direct effects on the relative abundance of *Chlorobi* in the *Artemisia nanschanica* group, while there was little difference in the relative abundance of *Chlorobi* in different plants. The reason for the inconsistent results between different studies may be because the current study used plants with rhizosphere soil, rather than surface soil. Both *Proteobacteria* and *Actinobacteria* with the highest relative abundance, and *Chlorobi* with the most significant differences in relative abundance are oligotrophic bacteria (Fierer et al., 2007; Ling et al., 2017). In the alpine steppe of northern Tibet, soil erosion is a serious problem, and the content of organic carbon is low (Cao and Wang, 2014). Poor soil is more suitable for the survival of oligotrophic bacteria, which is consistent with the results of this study.

At the genus level, only the average relative abundance of *Streptomyces* reached 10.55%, while the average relative abundance of other bacteria was <4%, indicating that *Streptomyces* was the dominant bacterium at the genus level (Figure 2B). *Streptomyces* is the largest taxa of bacteria and is widely distributed in nature (Barka et al., 2016), especially in soil. *Streptomyces* was previously reported to be the dominant bacterium in the alpine steppe environment in northern Tibet (Yin et al., 2016), which is consistent with the results of the current study. Previous studies have shown that *Streptomyces* is a chemoheterotrophic bacterium that not only inhibits the growth of pathogens through its own secondary metabolites (Rashad et al., 2015; Newitt et al., 2019) but is also instrumental in the turnover of organic matter, such as decomposition of recalcitrant carbon in deep soil (Fierer et al., 2007). In addition, the relative abundance of the genus *Micromonospora* was highest under grazing treatment and lowest under mowing treatment in the *Carex moorcroftii* group (Table 2). In the study of Della Monica et al. (2018), *Micromonospora* was considered a kind of bacteria that could promote or inhibit plant growth, and this may be the reason *Micromonospora* shows significant differences in different utilization patterns. Both *Streptomyces* and *Micromonospora* are actinomycetes, so it is necessary to strengthen further research on actinomycetes resources in this study area.

### Rhizosphere soil microbial diversity

The Shannon index of all samples in the current study was more than 0.97, and the Chao1 and Simpson indices were consistent within and between groups (Figure 5). Alpha diversity can reflect the number and abundance distribution of microbial communities from two aspects—richness and evenness (Li et al., 2020a), and the richness and diversity of soil microbial communities are often affected by the heterogeneity of vegetation and soil nutrients (Huang et al., 2021). However, in different grassland utilization patterns in the current study, there was almost no significant difference in rhizosphere bacterial community alpha diversity among all groups; the exception was the significant difference in the Shannon index of the *Artemisia nanschanica* group. These observations are consistent with those of a study conducted in Oxfordshire in southeast England (French et al., 2017), where bacterial diversity and specific beneficial taxa were unaffected by changes in land use. In addition, Zhang et al. (2019a) found that grazing did not have a significant effect on the rhizosphere and non-rhizosphere soil bacterial community structure in the Inner Mongolia Plateau. However, Bai et al. (2022) recently reported that long-term mowing significantly affected soil microbial community structure and composition. This was not reflected in the current study, which may be related to the scale of mowing time.

Overall, one of the reasons for the similarity of microbial alpha diversity in rhizosphere soil under different grassland utilization patterns may be driven by the survival strategies of soil microorganisms. For example, a study of the geographical pattern of Australian actinomycetes found that 32% of the bacteria were widely distributed in mainland Australia at the operational taxonomic unit (OTU) level, indicating that actinomycetes have a strong ability to disperse (Araujo et al., 2020). Jiang et al. (2021) reported that the similarity of soil bacterial community alpha diversity was due to limited soil nutrients. In the experimental area of the current study, the soil conditions have been very poor (Cao and Wang, 2014), and none of the treatments (grazing, mowing, or enclosing) had an obvious effect on the alpha diversity of rhizosphere soil bacterial community. This was verified in the study of Salles et al. (2004), demonstrating that previous land use is the predominant factor affecting the microbial community composition and that the influence of plant species on microbial community structure is less than that of the land-use history. The experimental area of the present study has been used as a grazing area, and its soil microbial communities are quite stable; thus, the area can withstand the impact of short-term mowing or enclosing.

The non-parametric (Kruskal–Wallis) test between species at the phylum level showed that there was almost no significant difference in plant rhizosphere microorganisms under different treatments (Table 1). NMDS (Figure 6) and MRPP (Table 3) confirmed this finding. Differences in rhizosphere soil microorganisms were greater among the three plants (groups) than differences within a group, but not all differences were significant. Differences in rhizosphere microorganisms among different plant species are expected, but the absence of a significant difference indicates that plant species had a weak effect on rhizosphere microbial communities and cannot be a predominant explanatory factor. A study in the Netherlands supported these findings (Kielak et al., 2008). Furthermore, the factors affecting rhizosphere soil microbial diversity are diverse and do not only comprise plant types and grassland utilization patterns but also include soil types and other factors. For example, laboratory control experiments on *Arabidopsis thaliana* revealed that rhizosphere microorganisms were strongly affected by soil type (Lundberg et al., 2012) and stage of development (Yuan et al., 2015). Future research should consider other factors to identify those that lead to, or best explain, differences or lack of differences in rhizosphere soil microorganisms.

### Microbial interaction network

Soil microorganisms can survive in complex ecological networks with different types of symbiosis, competition, predation, partial benefit, or partial pest symbiosis (Faust and Raes, 2012). The co-occurrence patterns of a soil microbial community not only facilitate understanding of microbial interactions from a new perspective (Ma et al., 2016) but can also explain the spatial niche of microorganisms (Freilich et al., 2018), which is of great significance for understanding biodiversity and community stability (Zhang et al., 2019c). In the current study, the network interaction of the *Artemisia nanschanica* group was more complex than that of the *Stipa Purpurea* group and the *Carex Moorcroftii* group in the plant group (Figure 7), and that of enclosing treatment was more complex than that of mowing and grazing treatments in the treatment group (Figure 8). Combining these two points led to the conclusion that the network interaction of rhizosphere soil microorganisms is more complex in an environment that is not easily affected by external factors. The plants under enclosing treatment are obviously unaffected by certain factors, such as livestock feeding and trampling activities, while *Artemisia nanschanica*, as a plant that livestock did not like to eat, had little effect on rhizosphere microbial communities even under grazing conditions. Du et al. (2020) noted that microbes can change their survival strategies to adapt to environmental changes and can cooperate with other microbial species in a nutrient-deficient environment. de Menezes and Banerjee (Banerjee et al., 2016; de Menezes et al., 2017) hypothesized that soil nutrients are key factors driving the interactions between microorganisms, and abundant resources can reduce negative interactions between microorganisms. In the current study, it can also be indirectly reflected that mowing and grazing will generally lead to a decrease in aboveground biomass, thus reducing the amount of soil nutrients imported from underground and increasing the retention of underground nutrient resources. Therefore, it can be speculated that under the conditions of mowing and grazing, the negative interactions of rhizosphere soil bacteria in the *Stipa purpurea* and *Carex moorcroftii* groups that livestock like to eat will be lower than that in the *Artemisia nanschanica* group, which is congruent with the experimental results of the present study. This finding needs further verification in the experimental area to account for the influence of soil factors. In addition, in all the network interactions, most of the core nodes correspond to *Proteobacteria* and *Actinobacteria*, and their relative abundance in the rhizosphere soil microbial community is also the highest among all detected phyla. Therefore, as the most important key bacterial taxa in the alpine steppe of northern Tibet, *Proteobacteria* and *Actinobacteria* play a crucial role in maintaining the stability of the microbial community.

## Conclusion

In general, three main conclusions can be drawn from the experimental area of the current study. (1) *Proteobacteria* and *Actinobacteria* are the most important key bacterial taxa in the alpine steppe of northern Tibet and play a crucial role in maintaining stability of the microbial community. (2) The strong dispersion ability of some bacteria and the close distance of the sample sites mean plant species and grassland utilization had little effect on the rhizosphere soil microbial community structure and diversity. (3) Grazing, mowing, and enclosing did not significantly change the rhizosphere soil microbial community structure and diversity but did change the network interactions of microorganisms. Findings from the study facilitate the understanding of the changes of microbial communities in plant rhizosphere soil under common grassland utilization patterns in northern Tibet and provide a theoretical basis for the restoration of degraded alpine grassland and the development of microbial function. Results from the study also demonstrate that the dominant bacteria in the rhizosphere soil are all oligotrophic microorganisms, which may be related to the poor soil in this region. Therefore, considering the important role of rhizosphere soil microorganisms in the turnover of plant soil nutrients, further metagenomics research on microbial functions is needed to establish the correlation with soil nutrients and provide a scientific basis for the formulation of ecological protection and restoration strategies.

## Data availability statement

The original data presented in the study are publicly available. This data can be found here: NCBI, under accession number: PRJNA838729.

## Author contributions

YY and LF conceived and designed the experiment. XLi collected the rhizosphere soil samples. LF analyzed all data, wrote the first draft of the manuscript, and revised the manuscript. LF, YY, XLu, XLi, and YL discussed the first draft. In addition, the natural science fund project applied by YY provided financial support for this research. All authors contributed to manuscript revision, read, and approved the final submitted version of the manuscript.

## Funding

This research was supported by the National Natural Science Foundation of China (41871049 and 41877338).

## Conflict of interest

The authors declare that the research was conducted in the absence of any commercial or financial relationships that could be construed as a potential conflict of interest. The reviewer WL declared a shared affiliation with the authors LF, XL, and YL to the handling editor at the time of review.

## Publisher's note

All claims expressed in this article are solely those of the authors and do not necessarily represent those of their affiliated organizations, or those of the publisher, the editors and the reviewers. Any product that may be evaluated in this article, or claim that may be made by its manufacturer, is not guaranteed or endorsed by the publisher.
